# The long is not just a sum of the shorts: on time experienced and other times

**DOI:** 10.3389/fpsyg.2014.00516

**Published:** 2014-05-28

**Authors:** Jiří Wackermann

**Affiliations:** Empirical and Analytical Psychophysics, Institute for Frontier Areas of Psychology and Mental HealthFreiburg im Breisgau, Germany

**Keywords:** time experience, time perception, biographic time, phenomenology, psychophysics

Psychology should be a positive, empirically informed science of human existence; that is, a science of human experience of and acting in the world, in their unity. Therefore, psychology has to take human experience seriously. What does it mean for “psychology of time”?

The term was coined more than a century ago (Nichols, 1891); but what “psychology of time” is or should be has never been defined with full clarity. Its core topic always has been “time perception”—originating in early psychophysical (Mach, 1865) and physiological (Vierordt, 1868) studies of “time sense”—with miscellaneous time-related topics (psychological, developmental, cultural) attached (Fraisse, 1963; Doob, 1971; Grondin, 2008). Recently, “recognition of the centrality of psychology of time” has been advocated (Hancock and Block, 2012), but what is that specific for psychology? Time is a universal concept used across scientific disciplines, from physics down to paleontology or archaeology. In physics, dynamical descriptions of the phenomena under study are essential, and thus dimension of time plays a fundamental rôle; yet there is no special chapter of physics named “physics of time” that could claim such a “centrality.” Psychology studies mental phenomena in their temporal course or relations—e. g., reaction times, choice times, dynamics of perception and cognition—but this still does not constitute a special “psychology of time.” As social and cultural beings, we act “in” time and with regard to temporal schedules: wide fields for psychological research, but why have a particular sub-discipline for that? Why not leave study of habitual latecomers to psychology of personality, and decision times to psychology of consumer behavior?

What makes an essential difference between events just occurring “in” time—motions of stars, geyser eruptions, heart contractions—and human acting “in” time is that we are *aware* of time. Or, more precisely: temporal characters, change, flux of world-states and bodily states, are integral part of our experience of the world and of our-selves in it.

The term “experience of time” is mostly used synonymously with that of “time perception,” though it may also cover experience of duration *plus* awareness of other, qualitative temporal characters (sensible present, past–future, etc.). I will use it in the more restrictive sense. Of course, “time perception” is a misnomer; “time is not an apple” (Woodrow, 1951). We do not perceive time; what we *do* perceive are events, occurring “out there” in the world, their temporal qualities and relations. Here we are interested not so much in performance of human subjects in “time perception” tasks but rather in the subjective experience as the primary basis upon which the notion and knowledge of time are constituted.

Consider a simple psychophysical experiment: a luminous stimulus of duration varied across several orders of magnitude (o. o. m.) is presented to the observer. For very short durations, from 1/1000 s up to about 1/100 s, only a flash of light of indefinitely short duration is seen. Variations of physical duration do not translate directly into experienced duration; it is rather the integral magnitude of the luminous sensation—its “volume,” so to speak—that changes. Conversely, changes in the exposure time can be counter-balanced by changing the luminous flux to obtain the same sensation magnitude (Talbot's law). From some critical duration on (~1/30 s), the percept gains an elementary temporal quality, so that the observer is able to distinguish between shorter and longer exposures, but its singular “flash-like” character is preserved. Finally, at exposure times of about 1/2 to 1 s, the onset and outset of the luminous appearance can be differentiated as two distinct events. From here on, the temporal extension of the stimulus is really *perceived* as duration.

The experiment illustrates existence of *regions* on the physical continuum of the control parameter (duration) delimited by more-or-less well defined *boundaries* (Wackermann, 2007). Stimuli from within the same region elicit percepts of the same quality; transgressing a boundary causes a transition to a different kind of experience. Descriptions applicable within one region cannot be transfered naïvely to another region, as exemplified by the brightness–duration interaction. This says that subjective experience is not deliberately “scalable” or decomposable into smaller elements. In our experiment, an extremely fast flash of light—say, a xenon-tube electric discharge in the o. o. m. of microseconds—will result in an irreducible sensory datum where the notions of “shorter” or “longer” cannot be applied meaningfully. It is not just “shorter” than in the o. o. m. of milliseconds; it is *qualitatively different* and thus *incommensurable*. In physics, 1 ms = 1000 × 1 μs; but one perceived “milliflash” does not consist of a thousand “microflashes.”

Perception of duration does not hold for deliberately long times, either. For example, in *duration reproduction* experiments with stimuli prolonged up to a few tens of seconds, the response curve—i. e., reproduced duration expressed as a function of the presented duration— progressively flattens, discrimination acuity decreases, and the observers become uncertain or unable of beholding the duration as a singular, indivisible experiential datum. This phenomenon finds a natural interpretation within the framework of the “lossy integration” model of neural representation of time (Wackermann and Ehm, 2006; Sysoeva et al., 2011): as the durations become comparable with the relaxation times of the lossy integrators, duration discrimination necessarily deteriorates. This is why we call this upper bound of “live” time experience the “horizon of reproducibility” (Wackermann, 2007). There is no sharp boundary,^1^ but the ability of duration representation as a unitary experiential datum definitely ceases somewhere in the o. o. m. of a few minutes. Alternative mechanisms may be involved and additional temporal cues invoked for a cognitive re-construction of longer durations.

This is *a fortiori* true for still longer times of hours, days, years. The multitude of conventional units alone indicates how time becomes fractioned and structured in a system of parallel overlapping time-scales, maintained by clocks and calendars. Clearly, it is the inability of human mind to keep a “measure of time” across longer intervals that enforced the invention of *external* time-keeping, chronometry, time reckoning (Whitrow, 1988; Birth, 2012). On these time-scales there is no “time experience”; these times are not “perceived,” only *known*. Also, external time-keeping allows us to refer even to events beyond the biological limits of our lives, and creates a kind of impersonal objectivity we associate with the order of world-time.

Touching the problem of human life-time: we should distinguish between biological time, seen from outside, and biographic time, seen from inside. Humans not only live but “conduct” their lives (Plessner, 1975), and are aware of times of their lives. This existential fact finds its psychological counter-part in the concept of “mental time travel,” that is, the ability “to mentally project themselves backwards in time to re-live, or forwards to pre-live, events” (Suddendorf and Corballis, 2007). It is a highly problematic metaphor, as the notion of “time travel” itself. Nonetheless, the catchy metaphor has made a career in the literature, and motivated research in comparative psychology, personality psychology, cognitive neuroscience, etc. (Suddendorf et al., 2009; Nyberg et al., 2010).

Now, representation of past events, or anticipation of future events refers explicitly to *biographic* time, consisting of (only partially ordered) biographic moments, episodes of personal relevance and existential importance, separated by indeterminate “time-spaces.” Some of past moments may be identified in terms of calendar time, but their “time indices” are more often given by a network of logical and material causalities, social circumstances, etc. The content of those episodes may be “experienced” (memory recall, anticipating imagination), but not their times; these are accessible only by cognitive (re)construction.

By contrast, experience of temporal characters of perceivable events (for short: “time perception”) happens on a “local timescale”: in the range (approximately) from 10^0^ s to 10^2^ s (Wackermann, 2007), i. e., of about two decadic o. o. m. A unique temporal quality specific for this domain is *duration*, as a measure of “temporal distance” between distinct events and sub-events. Only in this relatively narrow domain we can speak properly of “experience of time,” or “time experienced.”

These are two different orders of different kinds of events, or plainly: *two different times*. Biographic time is *not* a sum of chunks of experiential time; and conversely, experienced durations are *not* measures of fragments of biographic time. It is not only a matter of different time-scales: the long does not result from summation or multiplication of the short. The two orders of times are mutually irreducible and thus *incommensurable*. Of course, we can map both orders of time, those “perceived” and those “known,” onto the continuum of physical world-time. The difference of time scales does not play any rôle, since physical time is arbitrarily scalable. In physics, we can measure times of planetary motions by times of light-wave oscillations, or *vice versa*—not so in the realm of human experience^2^. The mapping onto a common background of world-time obscures or eliminates experiential content of the originals and the essential difference between them. Projections of two things onto a photographic plate give one fused image, but this does not make the two originals to be one thing!

Psychologists aiming at a unified “psychology of time” may be mislead by the concept of universal time of physics, applicable across all time-scales, and search for its analogy in the mental domain. Although they recognize differences between time of subjective experience and the objective clock-time—evergreen of popular “psychologies of time” since ever—they adhere to the idea of a unitary (though not inter-individually identical) order of mental or biographic events, arranged along a continuum of subjective time indices: “psychological time.” I will not reiterate my earlier critique of this concept (Wackermann, 2008); may it suffice to say that there is no necessity for the construct of “psychological time” and no compelling evidence for it. In fact, phenomenology of our experience of time speaks rather *against* it.

Once again: experience matters. We should observe the very structure and texture of experience precisely and adjust our conceptual schemes and theories to what is observed—not the other way round. Findings from experimental or clinical neuroscience may provide novel insights (Fingelkurts et al., 2010; Nyberg et al., 2010; Østby et al., 2012) but we should be aware that these are only *supplementary* data, informing but not determining our concepts. Regarding arguments from neural correlates and, particularly, conceptual unifications based on “shared networks”: there is only one brain for all kinds of mental functioning; resources are limited, so functional re-use is likely and probably necessary. The same applies, *mutatis mutandis*, to studies with neurological patients (El Haj et al., 2013).

The qualitative difference between “time experienced” and “time known” is evident and, in my view, unsurmountable. Instead of a forceful unification, a theory adequate to the structure of human experience should acknowledge and further elaborate this duality of times. How this can be done—this would be a subject for another essay. Here a few scarce remarks must suffice:

One-dimensional time is not an untouchable dogma; it is only a special feature of the arithmetic *model of time* employed in physics and public chronometry. We may think of the two times, biographic time and experienced time, not as two scales imposed on the same dimension but as two orthogonal dimensions (Figure 1). The *co-presence* of events within the horizon of actual presence is represented by the vertical, “depth” dimension, while the order of successive but essentially discontinuous episodes is represented by the horizontal dimension. It is only *post hoc*, in chronometric reconstructions, that both dimensions are collapsed and aligned with the continuum of the physical world time. Unlike popular metaphors of “passage of time,” “time flow” or point-like “now” sliding along the time axis, our picture suggests a different kinetic metaphor: a transversal wave in a stationary medium, where the “local motions” of the medium are not identical with the “global motion” (i. e., wave propagation) but orthogonal to it.

**Figure 1:**
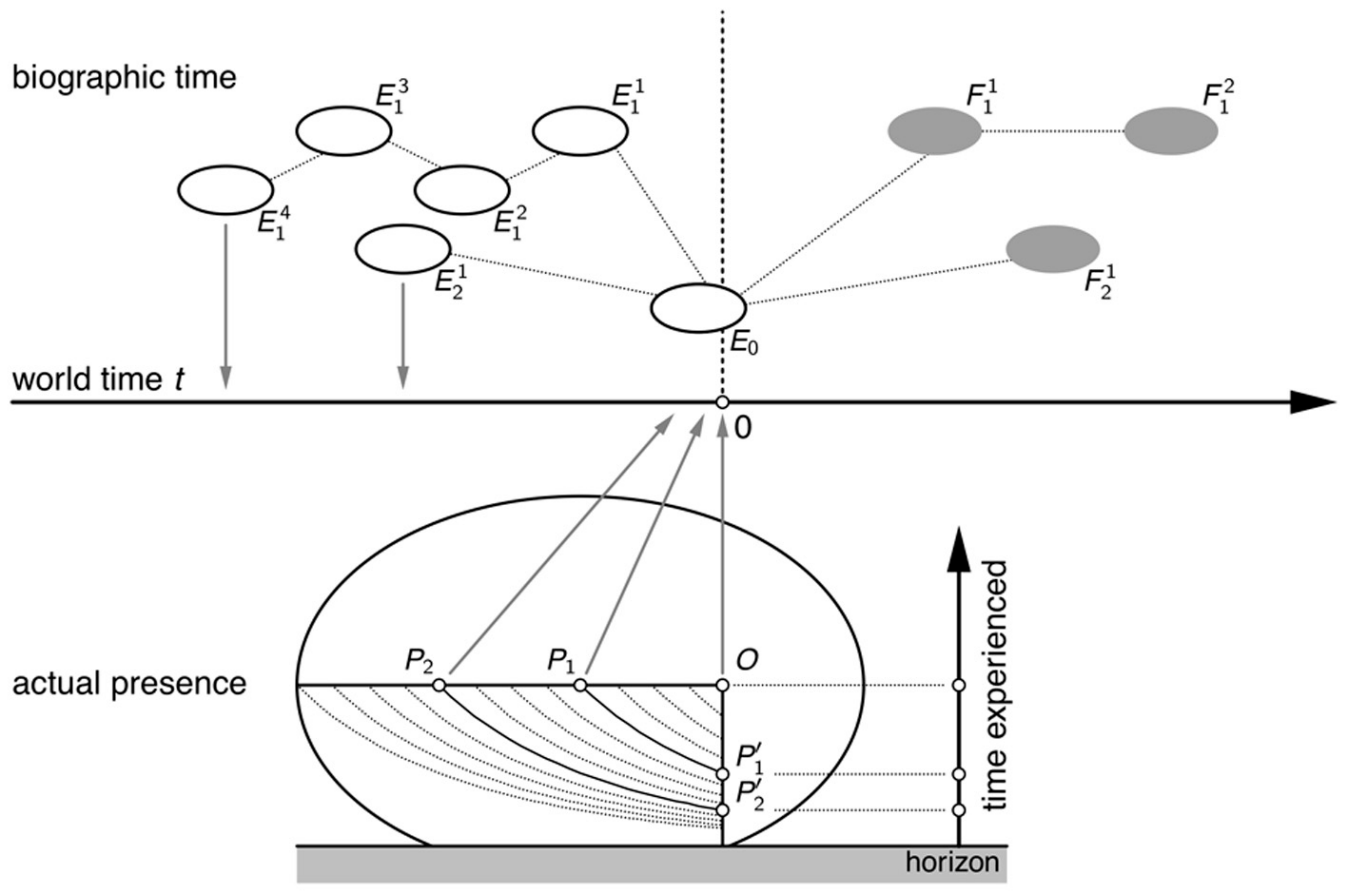
**Two dimensions of time**. Upper part: biographic time consists of passed (*E*^*n*^_*i*_) or anticipated (*F*^*n*^_*i*_) episodes, connected by logical or material dependencies (dotted lines) and thus partially ordered. Lower part: the actually present episode, *E*_0_, comprising subsequent events *P*_1_, *P*_2_, … that are co-present (*P*′_1_, *P*′_2_, …) to the observer's consciousness at *O* and ordered along the dimension of experienced time (vertical arrow). The two systems of events, inter-episodic (biographic) as well as intra-episodic (actual experience), are cognitively projected (gray arrows) onto the dimension of physical world time (horizontal arrow) which serves as a common reference for all time-orders and time-scales. Note: The two-dimensional map *P*_*n*_ → *P*′_*n*_ is essentially Husserl's (1928) “diagram of time,” modified in order to illustrate the non-linear metric of subjective duration and the finite horizon of time experience (cf. Wackermann, 2005, p. 201).

## Conflict of interest statement

The author declares that the research was conducted in the absence of any commercial or financial relationships that could be construed as a potential conflict of interest.

## Footnotes

^1^If our interpretation in terms of “lossy integration” is correct, the fuzziness of the upper boundary may be explained by inter-individual differences (Sysoeva et al., 2010) and intra-individual state-dependence (Späti, 2005; Wackermann et al., 2008) of the relaxation times of the integrators.

^2^In the model of time as arithmetic continuum, time indices of instants and time intervals between instants are naturally connected by arithmetic operations, and so we easily forget the generic difference between the indices (“when-times”) and durations (“between-times”). In subjective experience, however, these are two different things!
